# RP11-495P10.1 promotes HCC cell proliferation by regulating reprogramming of glucose metabolism and acetylation of the NR4A3 promoter via the PDK1/PDH axis: RP11-495P10.1 promotes HCC cell proliferation

**DOI:** 10.3724/abbs.2023242

**Published:** 2023-10-30

**Authors:** Chi Liu, Jie Shi, Zhengyuan Jiang, Shan Jiang, Yuan Wu, Dongqian Peng, Jiebing Tang, Linchi Guo

**Affiliations:** 1 General Medicine People’s Hospital of Ningxia Hui Autonomous Region Yinchuan 750000 China; 2 Department of Biochemistry & Molecular Biology Harbin Medical University Harbin 150000 China; 3 Department of Gastrointestinal Medical Oncology Harbin Medical University Cancer Hospital Harbin 150086 China; 4 Department of Endocrinology and Geriatrics Affiliated Renhe Hospital of Sanxia University Yichang 443000 China; 5 Department of Anatomy and Histology School of Preclinical Medicine Chengdu University Chengdu 610000 China

**Keywords:** HCC, RP11-495P10.1, PDK1, glycometabolism, NR4A3

## Abstract

The incidence and related death of hepatocellular carcinoma (HCC) have increased over the past decades. However, the molecular mechanisms underlying HCC pathogenesis are not fully understood. Long noncoding RNA (lncRNA) RP11-495P10.1 has been proven to be closely associated with the progression of prostate cancer, but its role and specific mechanism in HCC are still unknown. Here, we identify that RP11-495P10.1 is highly expressed in HCC tissues and cells and contributes to the proliferation of HCC cells. Moreover, this study demonstrates that RP11-495P10.1 affects the proliferation of HCC by negatively regulating the expression of nuclear receptor subfamily 4 group a member 3 (NR4A3). Glycometabolism reprogramming is one of the main characteristics of tumor cells. In this study, we discover that RP11-495P10.1 regulates glycometabolism reprogramming by changing the expression of pyruvate dehydrogenase kinase 1 (PDK1) and pyruvate dehydrogenase (PDH), thus contributing to the proliferation of HCC cells. Furthermore, knockdown of RP11-495P10.1 increases enrichment of H3K27Ac in the promoter of NR4A3 by promoting the activity of PDH and the production of acetyl-CoA, which leads to the increased transcription of NR4A3. Altogether, RP11-495P10.1 promotes HCC cell proliferation by regulating the reprogramming of glucose metabolism and acetylation of the NR4A3 promoter via the PDK1/PDH axis, which provides an lncRNA-oriented therapeutic strategy for the diagnosis and treatment of HCC.

## Introduction

Hepatocellular carcinoma (HCC) is one of the most serious malignant tumors in the world today and is the leading cause of cancer-related deaths [1]. The available treatment options for HCC are limited, and the effect is poor [2]. Therefore, an in-depth study of the occurrence and development of HCC is essential for formulating a reasonable diagnosis and treatment.

In recent years, the role of long noncoding RNAs (lncRNAs) in HCC has received increasing attention from researchers [ 3– 5]. LncRNAs are RNAs with lengths greater than 200 bp that do not encode proteins, and some of them encode short peptides [6]. LncRNA is not only an intermediary between DNA and protein but also a vital component in cell function [7]. A number of studies have shown that a variety of lncRNAs play vital roles in HCC. For example, HULC is a highly specific oncogenic lncRNA that can promote the development of HCC [8]. lncRNA DANCR contributes to the stem cell characteristics of HCC by downregulating the expression of CTNNB1 [9]. LncRNA LIMT contributes to sorafenib chemoresistance via regulation of miR-665 and epithelial to mesenchymal transition in HCC cells [10]. LncRNA RP11-495P10.1 (also called AC245100.4) is transcribed from chromosome 1q21.2, with a transcript length of 1555 bp, which is closely associated with the progression of prostate cancer [ 11, 12]. However, the specific mechanism of RP11-495P10.1 in HCC is still unclear.

One of the characteristics of tumor cells is metabolic changes [13]. Interestingly, tumor cells tend to generate energy through glycolysis rather than relying on mitochondrial oxidative phosphorylation, which is called the Warburg effect [14]. Glucose metabolism is often widely reprogrammed in the progression of cancer cells [15]. LncRNAs are also involved in the reprogramming process of glucose metabolism. For example, lncRNA MALAT1 controls cancer glucose metabolism and progression by upregulating the MYBL2-mTOR axis [16]. LncRNA HCG11 promotes 5-FU resistance in colon cancer cells by reprogramming glucose metabolism by targeting the miR-144-3p-PDK4 axis [17]. LncRNA GAL promotes colorectal cancer liver metastasis by regulating GLUT1-mediated glucose metabolism [18]. Therefore, whether RP11-495P10.1 regulates the reprogramming of glucose metabolism in HCC still needs to be investigated.

Nuclear receptor subfamily 4 group a member 3 (NR4A3, also known as NOR1) belongs to the orphan nuclear hormone receptor superfamily [19], which is a nuclear receptor and a transcription factor involved in various cell metabolism and tumor suppression processes [ 20– 22]. Furthermore, NR4A3 has been proven to be regulated by lncRNAs. For example, lncRNA LINC00467 inhibits NR4A3 posttranscriptionally by interacting with NR4A3 mRNA to form double-stranded RNA [23]. LncRNA AC245100.4 inhibits NR4A3 transcription by regulating STAT3 enrichment in the NR4A3 promoter region [11]. LncRNA BRE-AS1 physically binds STAT3, reduces the binding of STAT3 to the promoter of NR4A3, relieves the repression of NR4A3 caused by STAT3, and upregulates NR4A3 expression [24]. However, it is unclear whether NR4A3 is regulated by RP11-495P10.1 in HCC cells.

In this study, we intend to explore the role of RP11-495P10.1 and its downstream target gene NR4A3 in the proliferation of HCC cells. Furthermore, we will explore the mechanism of RP11-495P10.1 in HCC cell proliferation by reprogramming glucose metabolism and acetylation of the NR4A3 promoter.

## Materials and Methods

### Clinical tissue chips and chemicals

This project was approved by the Ethics Committee of People’s Hospital of Ningxia Hui Autonomous Region (approval number 2020-NZR-008). Chips containing 30 pairs of HCC tissues and their adjacent tissue samples were obtained from Outdo Biotech (HLivH060PG02; Shanghai, China). qRT-PCR assay was performed to evaluate the expression of RP11-495P10.1 and NR4A3 in HCC. Acetylation inhibitor C646 and PDK inhibitor JX06 were obtained from MCE company (Monmouth Junction, USA).

### Cell culture

All HCC cell lines (Huh7, HepG2 and Hep3B) and the human hepatic cell line LO2 were obtained from the Cell Bank of the Chinese Academy of Sciences (Shanghai, China). Cells were cultured in DMEM (Meilunbio, Dalian, China) supplemented with 10% FBS (Sigma-Aldrich, St Louis, USA) and 1% penicillin and streptomycin (Gibco, Carlsbad, USA) at 37°C under 5% CO _2_.

### Plasmid construction and cell transfection

The pcDNA3.1 vector containing RP11-495P10.1 and NR4A3 was synthesized by GENEWIZ (Suzhou, China). si-RP11-495P10.1 (5′-GUCCAGCUGUAUAAUGAAATT-3′), si-NR4A3 (5′-CCCUGGUAGAACUGAGGAATT-3′) and si-NC (5′-UUCUCCGAACGUGUCACGUTT-3′) were synthesized by GenePharma (Shanghai, China). sh-RP11-495P10.1 (5′-GCAAGGACTTGTAATCAAGAT-3′) and sh-ctrl (5′-TTCTCCGAACGTGTCACGT-3′) were obtained from RIBOBIO (Guangzhou, China). Before transfection, HepG2 and Hep3B cells were seeded in small dishes and cultured for 24 h. Subsequently, cells were transfected with siRNAs or plasmids using Lipofectamine 3000 (Invitrogen, Carlsbad, USA) according to the manufacturer’s instructions. Subsequent gain- and loss-of-function studies of RP11-495P10.1 were conducted 24 h after transfection.

### RNA extraction and real-time quantitative PCR (qRT-PCR)

TRIzol RNA isolation reagent (Takara, Beijing, China) was used to extract total RNA. The PrimeScript RT reagent Kit with gDNA Eraser (Takara, Beijing, China) was used for reverse transcription. qRT-PCR was performed on an ABI Prism 7500 PCR instrument with an SYBR Select Supermix kit (Thermo Fisher Scientific, Waltham, USA). The condition for reactions was as follows: 15 min initial incubation at 95°C, followed by 45 amplification cycles of denaturation at 94 °C for 10 s, annealing at 60°C for 30 s, and extension at 72°C for 20 s. The relative expression of RNA was calculated using the 2 ^‒ΔΔCt^ method. All primer sequences can be found in Supplementary Table S1.

### Western blot analysis

Cells were lysed in RIPA buffer (Beyotime, Shanghai, China) containing protease inhibitors (Sigma‑Aldrich), and the BCA Protein Assay Kit (Thermo Fisher Scientific) was used to quantify the concentration of proteins. Cell extracts were separated by 10% SDS-PAGE, transferred to NC membranes (Sigma‑Aldrich) and blocked in QuickBlock Blocking Buffer (Beyotime) for 10 min at room temperature. The membranes were incubated with primary antibodies against NR4A3 (Abcam, Cambridge, UK), PDK1 (Cell Signaling Technology, Danvers, USA), cyclinD1 (Cell Signaling Technology), and PCNA (Abcam) or GAPDH (Cell Signaling Technology) at 4°C overnight. After three washes with PBS, the membranes were incubated with HRP-conjugated secondary antibodies for 1 h at room temperature. Finally, the immunoblots were detected with BeyoECL Plus (Beyotime).

### Cell counting kit-8 (CCK-8) assay

After transfection for 24 h, the cells were detached by trypsin and fully dispersed. Next, the cells were counted and inoculated in 96-well plates. Ten microliters of CCK-8 solution (Sigma‑Aldrich) was added to each well and incubated for 2 h. Finally, the OD value was measured at 450 nm by using a microplate reader (800 TS; BioTek, Winooski, USA).

### Colony formation assay

After transfection for 24 h, the cells were detached with trypsin and fully dispersed. Next, the cells were counted and inoculated in 6-well plates. The cells were cultured for 2‒3 weeks and then fixed in formaldehyde and stained with crystal violet staining solution. The final count of colonies was completed using ImageJ software.

### Measurement of PDH activity, acetyl-CoA and lactate production and extracellular oxygen consumption rate (OCR)

After transfection for 48 h, the cells were ultrasonicated on ice. Then, the supernatant was recovered by centrifugation. Finally, PDH activity, concentrations of acetyl-CoA and lactate and OCR were measured using the pyruvate dehydrogenase (PDH) activity assay kit (Solarbio, Beijing, China), acetyl coenzyme A assay kit (Nanjing Jiancheng Bioengineering Institute, Nanjing, China), lactic acid assay kit (Nanjing Jiancheng Bioengineering Institute) and oxygen-sensitive time-fluorescent probes (Mito-Xpress, Luxcel Bioscience, Cork, Ireland), respectively. The results were normalized by protein concentration.

### Chromatin immunoprecipitation (ChIP)

A simple-ChIP Enzymatic Chromatin IP kit (Cell Signaling Technology) was used to detect the enrichment of H3K27Ac in the NR4A3 promoter. Anti-H3K27Ac (Cell Signaling Technology) was used in the ChIP assay, and goat anti-rabbit IgG (Cell Signaling Technology) was used as a negative control.

### Nude mouse xenograft model

‍All animal experiments were approved by the Human Ethics Committee of People’s Hospital of Ningxia Hui Autonomous Region (Approval number 2020-NZR-008). The experimental protocol was established according to the ethical guidelines of the Helsinki Declaration (Approval number 2020-NZR-008). All BALB/c nude mice were purchased from Beijing Vita River Experimental Animal Technology Co., Ltd (Beijing, China). Approximately 1×10 ^6^ sh-RP11-495P10.1 or sh-ctrl HepG2 cells were resuspended in media and then subcutaneously implanted in the flanks of male BALB/c nude mice (4–6 weeks old, *n*=3 per group). The mice were raised in sterile conditions. The appearance of a subcutaneous mass approximately one week after inoculation indicated successful inoculation. Tumor volumes were measured with a calliper and calculated using the standard formula: V=(length×width ^2^)/2. The mice were euthanized by dislocation of the cervical spine after five weeks. Tumor tissues were collected, weighed and subjected to immunohistochemical (IHC) analysis of NR4A3 expression.

### IHC staining

Mouse tumor tissues were fixed in 4% (w/v) paraformaldehyde, dehydrated in graded ethanol, and embedded in paraffin. Immunohistochemical staining was applied on 4 μm-thick slices of mice tumor tissues. The sections were stained anti-NR4A3 (Abcam, Cambridge, UK) was carried out according to the manufacturer’s instructions.

### Statistical analysis

Statistical analysis was performed with GraphPad Prism 7.0. (GraphPad Software, La Jolla, USA). All quantitative experiments were repeated in triplicate, and data were presented as the mean±SD. Student’s *t*-test or one-way ANOVA was applied to determine statistical significance. *P*<0.05 was considered statistically significant.

## Results

### RP11-495P10.1 was highly expressed in HCC tissues and cells and was closely related to the proliferation of HCC cells

**Figure 1:**
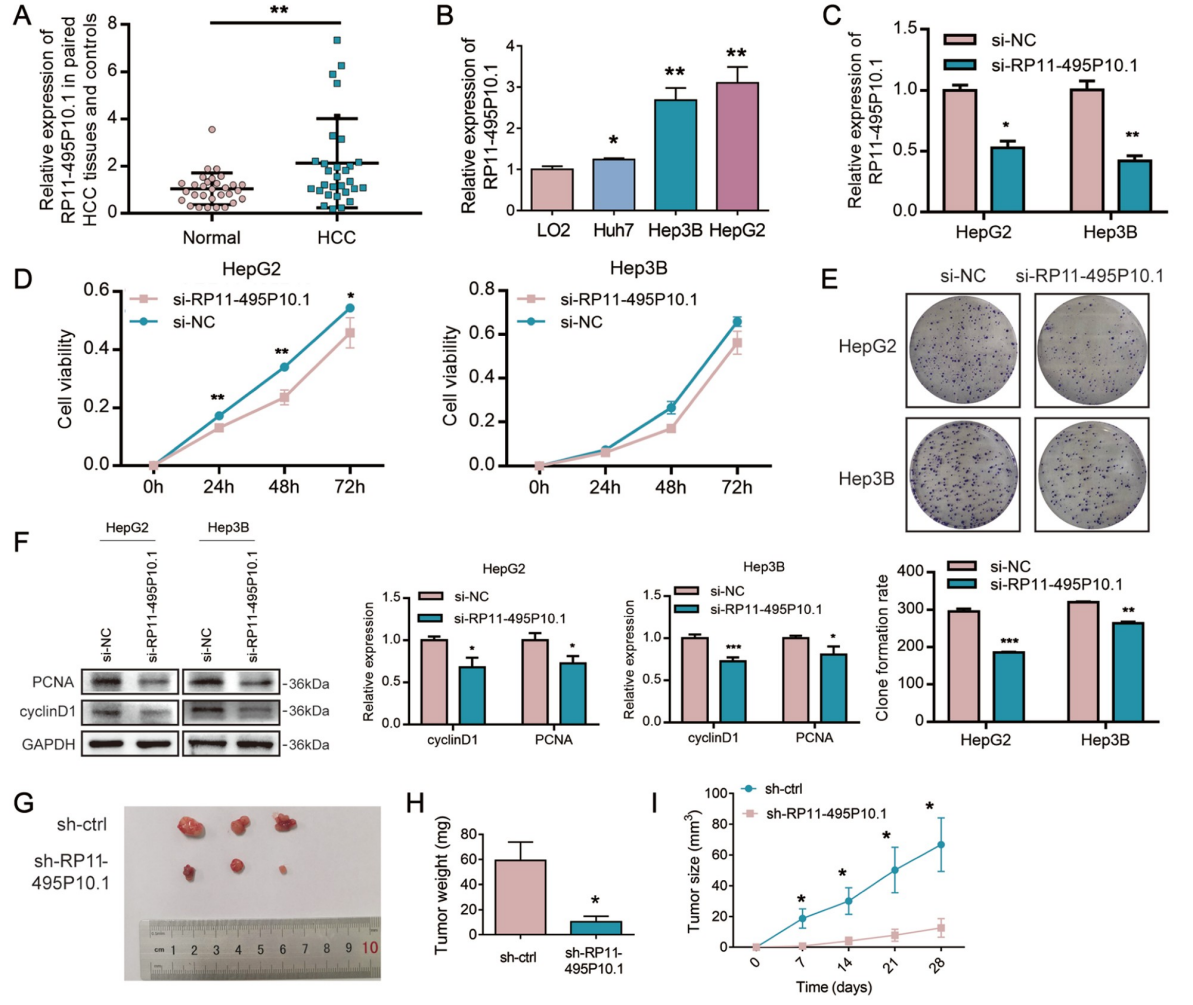
Figure 1 RP11-495P10.1 was highly expressed in HCC tissues and cells and was closely related to the proliferation of HCC cells (A) The expression of RP11-495P10.1 in 30 pairs of HCC tissues. (B) The mRNA expression of RP11-495P10.1 in HCC cells (Huh7, Hep3B and HepG2) and normal hepatic cells (LO2). (C) The interference efficiency of RP11-495P10.1 was measured by qRT-PCR in HepG2 and Hep3B cells. (D,E) CCK-8 and colony formation assays were performed to detect the proliferation of HCC cells. (F) CyclinD1 and PCNA expression in HepG2 and Hep3B cells with RP11-495P10.1 knockdown was detected by western blot analysis. (G) A total of 106 HepG2 cells with sh-RP11-495P10.1 or sh-ctrl were subcutaneously injected into mice for 4 weeks. Individual tumors were dissected and digitally photographed. (H) The volume of subcutaneous tumors. (I) The weight of subcutaneous tumors. *P<0.05, **P<0.01 and ***P<0.001 vs Normal, LO2, si-NC, sh-ctrl.

To explore the relationship between RP11-495P10.1 and HCC progression, we detected the expression of RP11-495P10.1 in 30 pairs of HCC clinical tissue specimens. The results showed that RP11-495P10.1 was highly expressed in HCC tissues ( Figure 1A). Next, we found that RP11-495P10.1 was highly expressed in Huh7, HepG2 and Hep3B cells compared with that in LO2 cells ( Figure 1B). The above results indicated that RP11-495P10.1 was closely related to the tumorigenesis of HCC. In malignant tumor cells, cell proliferation is often abnormally regulated and exhibits abnormal proliferation. Studies have shown that a variety of lncRNAs affect the occurrence and development of HCC by regulating downstream target genes [ 25– 27]. Therefore, to explore the effect of RP11-495P10.1 on cell proliferation in HCC cells, we first detected the interference efficiency of si-RP11-495P10.1 in HepG2 and Hep3B cells. The results showed that si-RP11-495P10.1 significantly decreased the expression of RP11-495P10.1 in HepG2 and Hep3B cells ( Figure 1C). We further detected cell proliferation by CCK-8 and colony formation assays in HepG2 and Hep3B cells with RP11-495P10.1 knockdown. The results showed that cell viability was significantly reduced and that the number and size of colonies were also significantly reduced by RP11-495P10.1 knockdown ( Figure 1D,E). Moreover, the expression of cyclin D1 and PCNA was also decreased by RP11-495P10.1 knockdown ( Figure 1F). In contrast, overexpression of RP11-495P10.1 promoted the proliferation of HCC cells ( Supplementary Figure S1A‒C). In addition, we further verified the role of RP11-495P10.1 *in vivo*. A total of 10 ^6^ HepG2 cells transfected with sh-RP11-495P10.1 or sh-ctrl were subcutaneously injected into mice for 4 weeks. The volume of subcutaneous tumors was measured every week. At the fourth week, subcutaneous tumors were dissected and digitally photographed ( Figure 1G). As shown in Figure 1H,I, the volume and weight of subcutaneous tumors were significantly decreased by sh-RP11-495P10.1. These results showed that RP11-495P10.1 promoted the proliferation of HCC cells.

### RP11-495P10.1 negatively regulated NR4A3, and overexpression of NR4A3 inhibited the proliferation of HCC cells

**Figure 2:**
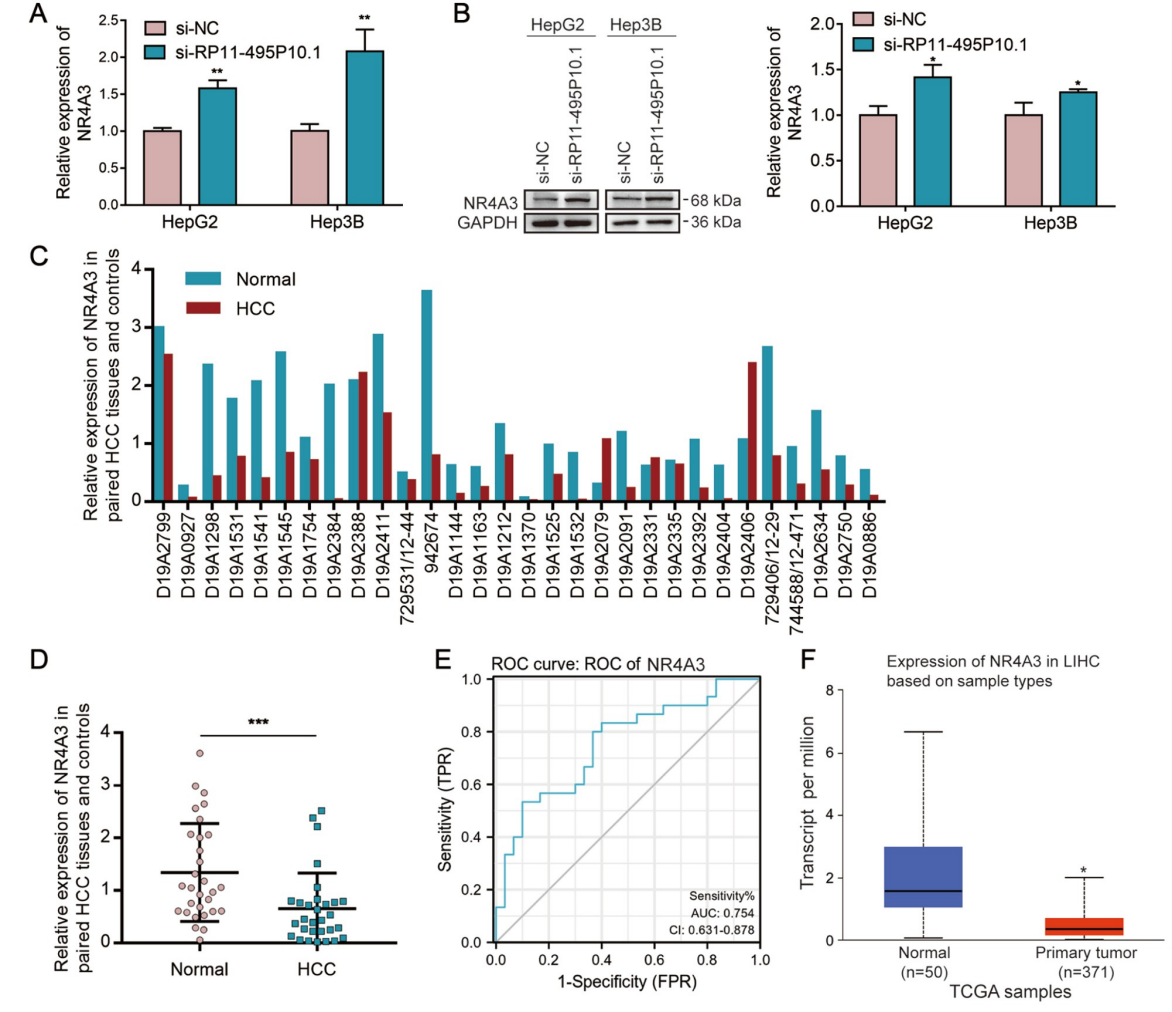
Figure 2 NR4A3 was negatively regulated by RP11-495P10.1 and expressed at low levels in HCC tissues (A,B) NR4A3 expression was tested by qRT-PCR and western blot analysis in HepG2 and Hep3B cells with RP11-495P10.1 knockdown. (C,D) NR4A3 expression in 30 pairs of chips was measured by qRT-PCR. (E) The ROC curve of NR4A3 in 30 pairs of chips. (F) NR4A3 expression in HCC tissues was obtained from UALCAN. *P<0.05, **P<0.01 and ***P<0.001 vs si-NC, Normal.

In our previous research on prostate cancer, we found that NR4A3 is one of the downstream target genes of RP11-495P10.1 [11]. To further verify that NR4A3 is the downstream target gene of RP11-495P10.1 in HCC cells, we detected NR4A3 expression by qRT-PCR and western blot analysis in HepG2 and Hep3B cells with RP11-495P10.1 knockdown. As shown in Figure 2A,B, NR4A3 expression was significantly increased by knockdown of RP11-495P10.1. Moreover, the results of IHC suggested that knockdown of RP11-495P10.1 decreased the expression of NR4A3 *in vivo* ( Supplementary Figure S2). Next, we used qRT-PCR to detect NR4A3 expression in 30 pairs of HCC tissues and adjacent normal tissues. The results showed that NR4A3 expression was downregulated in 26 HCC tissues (87%) compared with that in adjacent normal tissues ( Figure 2C,D). Moreover, the ROC curve of NR4A3 showed that the area under the curve was 0.754 (>0.5) ( Figure 2E). Similarly, TCGA data showed that the expression of NR4A3 was lower in HCC tissues than in normal liver tissues ( Figure 2F). These results indicated that NR4A3 may be a potential independent marker of HCC prognosis.

**Figure 3:**
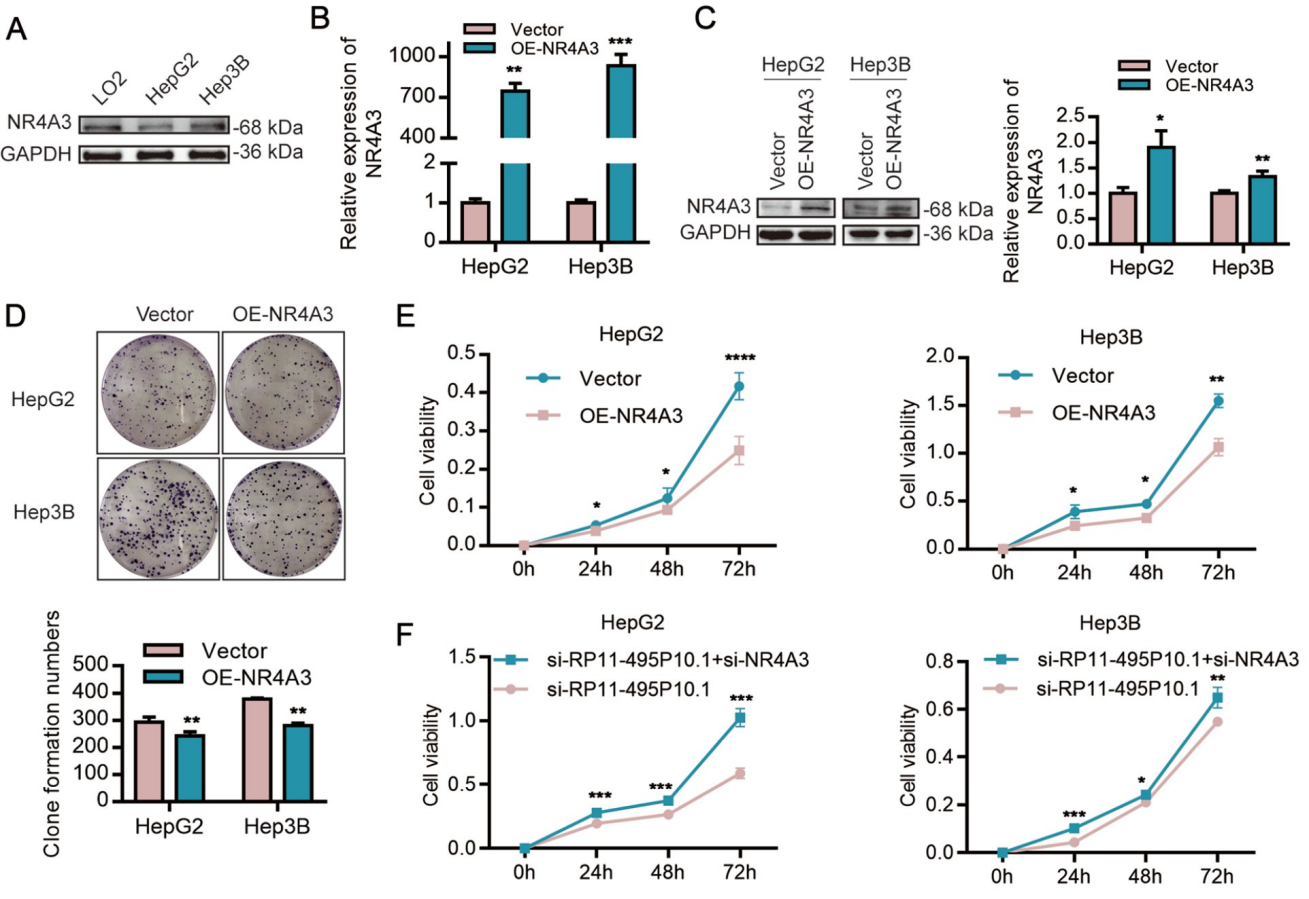
Figure 3 Overexpression of NR4A3 inhibited the proliferation of HCC cells (A) The expression of NR4A3 was detected by western blot analysis in HepG2, Hep3B and LO2 cells. (B,C) The overexpression efficiency of NR4A3 in HepG2 and Hep3B cells was verified by qRT-PCR and western blot analysis. (D,E) CCK-8 and colony formation assays were used to verify cell proliferation in HCC cells overexpressing NR4A3. (F) Rescue assays were used to evaluate the change in cell proliferation in HCC cells after co-transfection with si-RP11-495P10.1 and si-NR4A3. *P<0.05, **P<0.01 and ***P<0.001 vs Vector, si-RP11-495P10.1.

Previous studies have reported that NR4A3 can regulate the growth and proliferation of HCC cells [28]. To explore the role of NR4A3 in the proliferation of HCC cells, we first detected the expression of NR4A3 in HCC cells and LO2 cells. As shown in Figure 3A, low expression of NR4A3 was discovered in HCC cells compared with that in LO2 cells. We further constructed pcDNA3.1 (vector) and pcDNA3.1-NR4A3 (OE-NR4A3) recombinant plasmids and verified them in HepG2 and Hep3B cells ( Figure 3B,C). Then, colony formation and CCK-8 assays were performed to detect cell proliferation after overexpression of NR4A3. As shown in Figure 3D,E, overexpression of NR4A3 significantly decreased cell viability and the number and size of colonies in HepG2 and Hep3B cells ( Figure 3D,E). Next, to verify the role of RP11-495P10.1 in the proliferation of HCC through NR4A3, we co-transfected si-RP11-495P10.1 and si-NR4A3 into HepG2 and Hep3B cells and used CCK8 to detect cell proliferation. As expected, si-NR4A3 decreased the inhibition of cell proliferation by si-RP11-495P10.1 in HepG2 and Hep3B cells ( Figure 3F). These results suggested that NR4A3 was negatively regulated by RP11-495P10.1 and was closely related to the proliferation of HCC cells.

### RP11-495P10.1 regulated glycometabolism reprogramming by the PDK1/PDH axis

**Figure 4:**
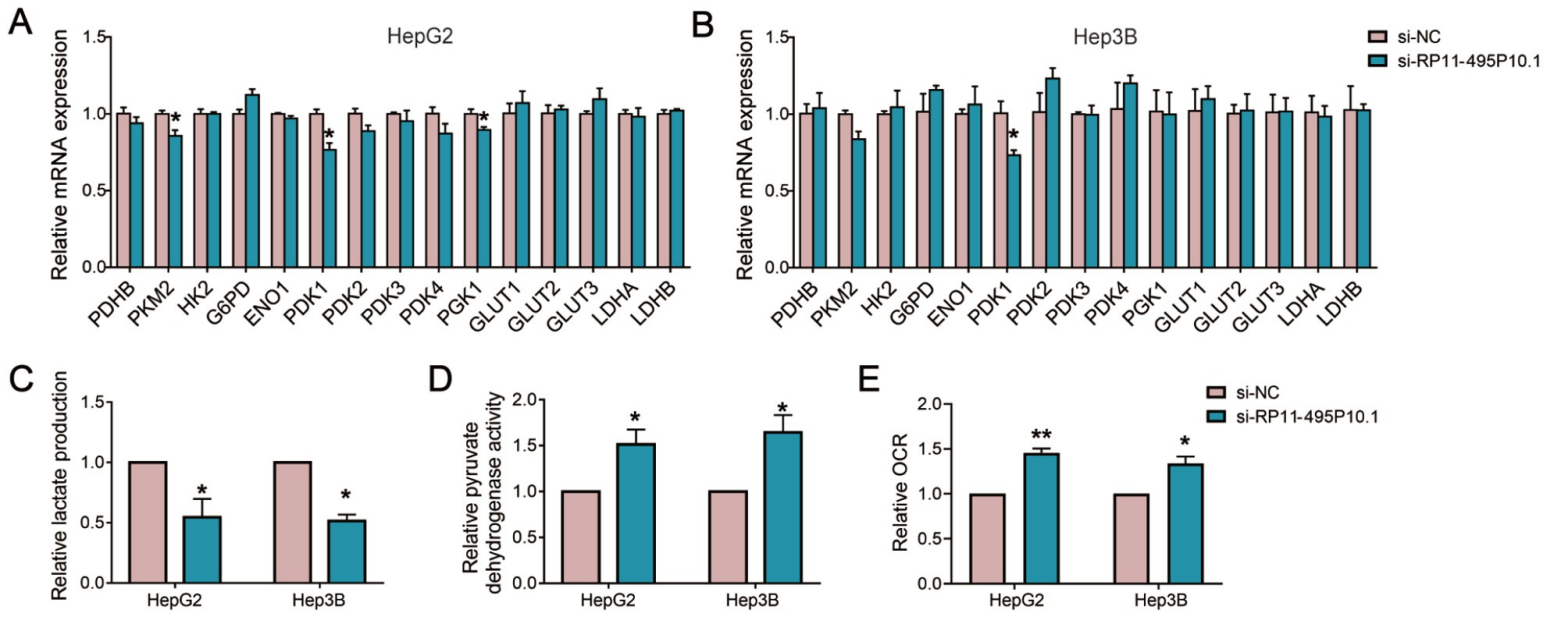
Figure 4 RP11-495P10.1 regulated glycometabolism reprogramming by PDK1 (A,B) The relative expression of key enzymes of glucose meta-bolismwas detected in HepG2 and Hep3B cells following RP11-495P10.1 knockdown by qRT-PCR. (C) Lactate production was detected in HepG2 and Hep3B cells with RP11-495P10.1 knockdown. (D) Pyruvate dehydrogenase activity was detected in HepG2 and Hep3B cells with RP11-495P10.1 knockdown. (E) Extracellular OCR was measured in HepG2 and HepG3B cells with RP11-495P10.1 knockdown. *P<0.05 vs si-NC.

Increasing evidence has shown that lncRNAs play a key role in the malignant progression of HCC through glycometabolism reprogramming [ 29, 30]. To investigate whether RP11-495P10.1 affects glucose metabolism in HCC cells, we detected the expression of key enzymes in glucose metabolism in HepG2 and Hep3B cells with RP11-495P10.1 knockdown by qRT-PCR. The results showed that RP11-495P10.1 significantly decreased the expression of PDK1 ( Figure 4A,B). Pyruvate dehydrogenase kinase 1 (PDK1) is an enzyme that inactivates mitochondrial pyruvate dehydrogenase (PDH), leading to the transformation of glucose metabolism from mitochondrial oxidation to glycolysis [31]. Generally, cancer cells increase glycolysis and metabolism and produce a large amount of lactate even in an aerobic environment [32]. Therefore, to understand the effect of RP11-495P10.1 on glucose metabolism in HCC cells, we first examined its effect on lactate production and PDH activity. The results showed that silencing of RP11-495P10.1 inhibited the production of lactate and increased PDH activity in HepG2 and Hep3B cells ( Figure 4C,D). Moreover, we detected extracellular OCR in HCC cells with RP11-495P10.1 knockdown, and the results showed that knockdown of RP11-495P10.1 resulted in an increase in oxygen consumption ( Figure 4E). These results demonstrated that knockdown of RP11-495P10.1 inhibited the glycolysis pathway and promoted the oxidative phosphorylation pathway, suggesting that knockdown of RP11-495P10.1 can regulate glycometabolism reprogramming in HCC cells.

Previous studies have confirmed that JX06 is a new inhibitor of PDK1. To further study the role of PDK1 in the reprogramming of glucose metabolism and the proliferation of HCC cells, we first detected the expression of PDK1 in Huh7 cells treated with 0.05 μM JX06. The results showed that JX06 significantly inhibited the expression of PDK1 in Huh7 cells ( Supplementary Figure S3A). Moreover, we found that JX06 increased PDH activity and inhibited the production of lactate ( Supplementary Figure S3B,C). To further study the role of PDK1 in reprogramming glucose metabolism and cell proliferation in HCC cells, we added JX06 to Huh7 cells. Interestingly, JX06 reversed the changes in PDH activity ( Supplementary Figure S4A) and lactate production ( Supplementary Figure S4B) induced by overexpression of RP11-495P10.1. In addition, JX06 reversed the promotion of RP11-495P10.1 overexpression on cell proliferation in Huh7 cells ( Supplementary Figure S4C). In conclusion, these results suggested that RP11-495P10.1 regulated the reprogramming of glucose metabolism in HCC cells through PDK1, which affected the proliferation of HCC cells.

### RP11-495P10.1 regulated the acetylation enrichment of the NR4A3 promoter through the PDK1/PDH axis

**Figure 5:**
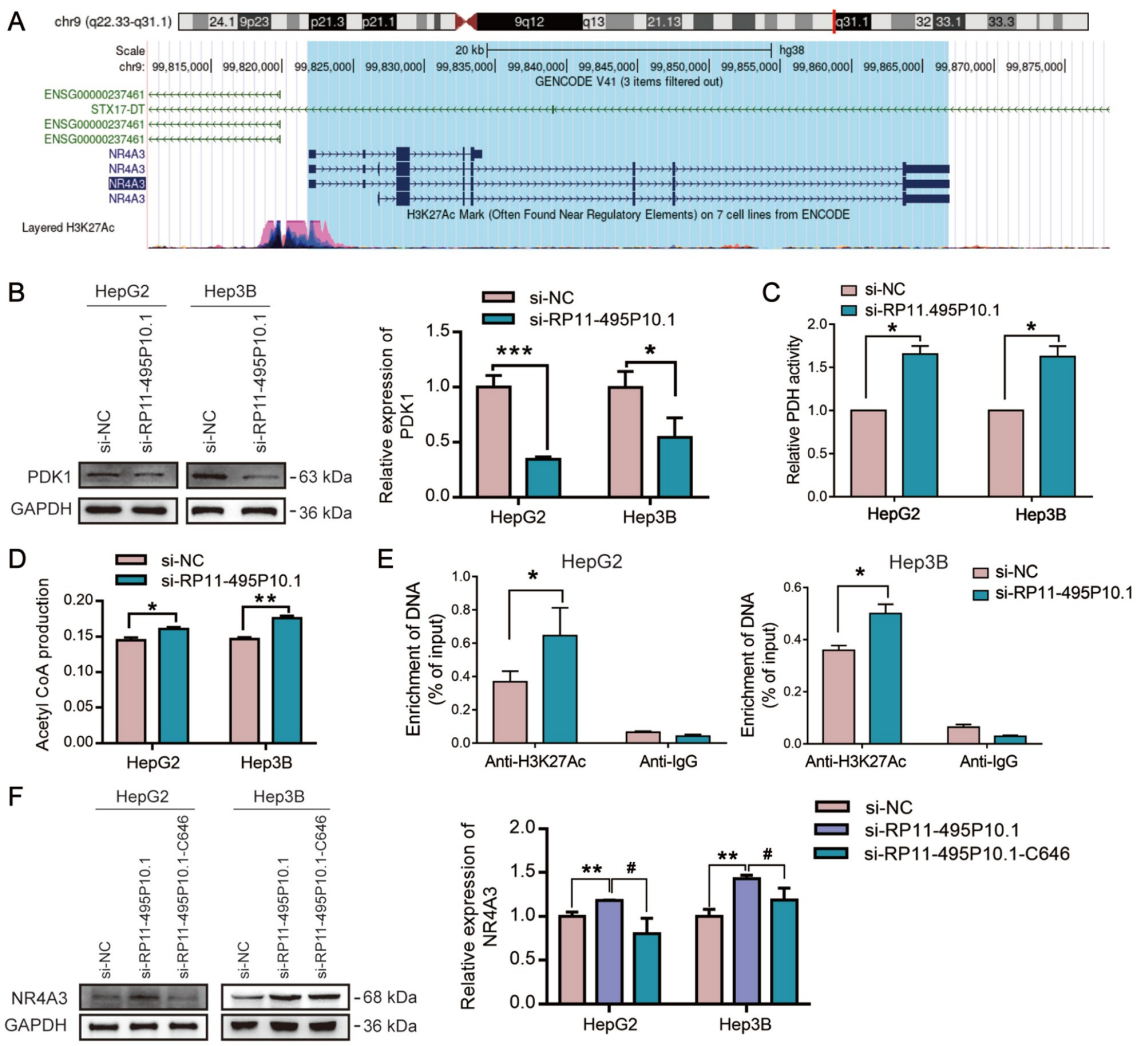
Figure 5 Knockdown of RP11-495P10.1 increased the acetylation enrichment of the NR4A3 promoter through PDH (A) H3K27Ac enrichment in the NR4A3 promoter was obtained from UCSC. (B) PDK1 expression was detected by western blot analysis in HepG2 and Hep3B cells with RP11-495P10.1 knockdown. (C) Detection of PDH activity in HepG2 and Hep3B cells with RP11-495P10.1 knockdown. (D) Detection of acetyl-CoA production in the nucleus of HepG2 and Hep3B cells with RP11-495P10.1 knockdown. (E) Enrichment of H3K27Ac in the promoter of NR4A3 was detected by ChIP in HepG2 and Hep3B cells with RP11-495P10.1 knockdown. (F) C646 (20 μM) was added to HepG2 and Hep3B cells with RP11-495P10.1 knockdown, and the expression of NR4A3 was detected by western blot analysis. *P<0.05, **P<0.01, ***P<0.001 vs si-RP11-495P10.1; #P<0.05 vs si-NC.

Studies have reported that the modification of histones neutralizes the electrical properties and leads to increased chromatin openness and upregulation of gene expression, while the modification of histone acetylation requires acetyl-CoA [33]. PDH is a key enzyme that catalyzes pyruvate to produce acetyl-CoA [34]. Therefore, we used UCSC ( http://genome.ucsc.edu/) to predict the degree of enrichment of histone acetylation in the NR4A3 promoter region. As shown in Figure 5A, the NR4A3 promoter region has a higher abundance of H3K27Ac acetylation sites. To investigate whether RP11-495P10.1 affects the activity of PDH by regulating the expression of PDK1, we first detected the expression of PDK1 by western blot analysis, and the results showed that knockdown of RP11-495P10.1 significantly decreased the expression of PDK1 ( Figure 5B). We further detected the activity of PDH. As shown in Figure 5C, the activity of PDH was highly increased by knockdown of RP11-495P10.1. Next, we detected changes in acetyl-CoA in the nucleus and found that acetyl-CoA was increased by knockdown of RP11-495P10.1 in the nucleus ( Figure 5D). It is worth noting that large amounts of acetyl-CoA may lead to histone acetylation and thus promote NR4A3 transcription; therefore, we further detected the enrichment of H3K27Ac in the promoter of NR4A3 in HepG2 and Hep3B cells. As expected, the enrichment of H3K27Ac in the promoter of NR4A3 was increased in HepG2 and Hep3B cells ( Figure 5E). These results indicated that RP11-495P10.1 could influence NR4A3 transcription. In addition, we added the acetylation inhibitor C646 to HepG2 and Hep3B cells with RP11-495P10.1 knockdown and detected NR4A3 expression by western blot analysis. As shown in Figure 5F, the acetylation inhibitor C646 reversed the promotion of NR4A3 transcription by si-RP11-495P10.1. These results indicated that si-RP11-495P10.1 activated PDH by inhibiting PDK1 expression and catalyzing the generation of acetyl-CoA, thus promoting the opening of NR4A3.

## Discussion

An increasing number of studies have shown that many functional lncRNAs are closely related to the malignant progression of HCC [35]. Here, we identified that RP11-495P10.1 was highly expressed in HCC tissues and cells and revealed the oncogenic role of RP11-495P10.1 in the proliferation of HCC cells. Mechanistically, RP11-495P10.1 regulates glucose metabolism reprogramming and acetylation of NR4A3 through PDK1, thereby affecting HCC cell proliferation. This research may provide new possibilities for the diagnosis and treatment of HCC.

The molecular mechanisms of lncRNAs are manifold [36]. Generally, lncRNAs can interact with DNA, proteins or RNAs to participate in the regulation of oncogenes or tumor suppressor genes in cancer [ 35, 37]. NR4A3 is one of the members of the solitary nuclear receptor superfamily [38] with a molecular weight of approximately 68 kDa, which has been shown to generally play tumor-suppressive roles in a variety of cancers [ 23, 39, 40]. In this study, we discovered that RP11-495P10.1 promotes the proliferation of HCC cells. Furthermore, the expression of NR4A3 was significantly increased by the knockdown of RP11-495P10.1, and overexpression of NR4A3 significantly inhibited the proliferation of HCC cells. Metabolic reprogramming is a biological characteristic acquired during the multistep process of tumor development [41]. Occasionally, tumor cells prioritize glycolysis to convert glucose to lactate to provide more metabolites for cell growth even when oxygen is adequate [42]. Recent research has shown that lncRNAs are involved in the reprogramming of energy metabolism by affecting associated enzymes and products. For example, it has been reported that lncRNA-SOX2OT promotes aerobic glycolysis in HCC via PKM2 regulation and activation and accelerates the occurrence and development of HCC [43]. Furthermore, lncRNA MSC-AS1 regulates aerobic glycolysis and promotes HCC incidence by inducing the expression of PGK1 [44]. Moreover, lncRNA SLC2A1-AS1 suppresses GLUT1 expression by inhibiting the STAT3/FOXM1 axis and influences aerobic glycolysis and HCC progression [45]. In this study, we found that knockdown of RP11-495P10.1 increased the activity of PDH, made pyruvate more inclined to produce acetyl-CoA than lactate and enhanced oxygen consumption. In other words, the existing results suggested that RP11-495P10.1 was related to glucose metabolism reprogramming in HCC cells. One of the characteristics of tumor cells is the Warburg effect. Even under aerobic conditions, tumor cells tend to use the glycolysis pathway rather than aerobic oxidation to generate energy [14]. The reason why tumor cells choose the glycolysis pathway rather than the oxidative phosphorylation pathway for energy production is that glycolysis can rapidly supply energy and provide more carbon sources. In this study, we found that knockdown of RP11-495P10.1 enhanced oxidative phosphorylation and weakened glycolysis in HCC cells, thus reducing the supply of carbon sources and the energy supply rate, leading to inhibition of cell proliferation.

Histone modification neutrally and electrically leads to increased chromatin openness and upregulation of gene expression, and the modification of histone acetylation requires acetyl-CoA [33]. The preliminary results of our research group showed that interference with RP11-495P10.1 could downregulate the expression of PDK1. There are four isoenzymes of PDK, among which PDK1 is widely expressed in all systems and shows strong sensitivity to all regulatory reactions [46], which can regulate the activity of PDH [47]. In this study, we found that knockdown of RP11-495P10.1 inhibited PDK1 expression and increased the activity of PDH, which led to an increase in acetyl-CoA in the nucleus. The increase in acetyl-CoA promoted the enrichment of H3K27Ac in the promoter of NR4A3. Finally, we discovered that histone acetylation inhibitors reversed the promoting effect of RP11-495P10.1 knockdown on NR4A3 transcription. In the final analysis, we demonstrated that knockdown of RP11-495P10.1 increased acetylation enrichment in the NR4A3 promoter region through the PDK1/PDH axis, which led to the activation of NR4A3 transcription. These results suggested that knockdown of RP11-495P10.1 upregulated the expression of NR4A3 through PDK1/PDH, thereby inhibiting HCC cell proliferation.

Unfortunately, although we found that RP11-495P10.1 is closely related to glucose metabolism in HCC, we did not explore it in depth. In this study, we mainly focused on the epigenetic regulation of NR4A3, while for research on RP11-495P10.1 and glucose metabolism, we will further explore this topic in the next study.

In conclusion, our study showed that RP11-495P10.1 is a new highly expressed and carcinogenic lncRNA in HCC. RP11-495P10.1 regulates glucose metabolism reprogramming and NR4A3 transcription through the PDK1/PDH axis, thereby affecting HCC cell proliferation. Mechanistically, knockdown of RP11-495P10.1 increases enrichment of H3K27Ac in the promoter of NR4A3 by increasing the activity of PDH and the production of acetyl-CoA, which leads to the increased transcription of NR4A3. Targeting RP11-495P10.1 or NR4A3 may be a potential treatment for HCC. Our findings contribute to a deepened understanding of lncRNAs in HCC tumor biology and provide new strategies for the diagnosis and treatment of HCC.

## Supplementary Data

Supplementary data is available at *Acta Biochimica et Biphysica Sinica* online.
